# Stomatitis from a Dental Stand-Point

**Published:** 1902-09

**Authors:** D. Genese

**Affiliations:** Baltimore, Md.


### STOMATITIS FROM A DENTAL STAND-POINT.[1]

BY DR. D. GENESE, BALTIMORE, MD.

The practice of dentistry of to-day calls for more than the knowledge of filling teeth and making dentures. The subject stomatitis, and its kindred complications, is as much of interest to the dental as to the medical practitioner.

The dental operations termed oral surgery cannot be undertaken if our patients are not in a proper physical condition to bear the manipulations needed. It is our duty to obtain the history of our patient pertaining to the conditions presented to us just the same as our medical *confrère* would when consulted by his patient. While dental lesions have their reflex action upon the nervous system, stomatitis has the same, and will often render a patient totally unfit to remain long in the dental chair.

Stomatitis, so intimately associated with organs of mastication and the oral cavity in general, should claim our earnest attention, and every dental practitioner should not fail to put into practice his medical training received at college and in daily practice, and give his patients the benefit of his experience instead of abandoning this part of his education for the exclusive work of filling teeth and making artificial ones.

Our energies to get at work often leads to a long period of nervous prostration of our patient, who may be suffering from stomatitis or gastritis, and the dental practitioner gets blamed for being the cause, whereas he would have avoided the delay and annoyance by a careful inquiry and a diagnosis of his patient's condition previous to commencing the dental operation.

When we are called upon to treat a case of facial neuralgia of long standing, our inquiries should be very searching, and we are sure to find some derangement of the stomach and digestive organs involved, as the following case will illustrate.

Mr. H., about fifty-five years old, had had some lower molars extracted some weeks previously to my seeing him; pain had not only continued, but increased in intensity, and large quantities of morphine had been administered without allaying the pain. His

[1] Read before the Academy of Stomatology, Philadelphia, February 25, 1902.

medical adviser sent for me to meet in consultation. His condition was serious,—moaning with pain; high fever; pulse 95; lips and tongue nearly black; fetid odor; and it looked like a case of typhoid. But the patient was conscious and able to answer questions, which helped the diagnosis. There had been great hemorrhage, and no doubt blood had been swallowed. The administrations of the morphine in large quantities had locked up all secretions, and constipation had existed for a week. Here evidently was a case of severe form of reflex action from the pneumogastric nerve. Calomel had been administered without effect, also bluet drops. I advised the use of glycerin in suppository form, one every fifteen minutes until relieved. It took eight sixty-five-minim suppositories to get the desired effect, and, to use the patient's words, he said he could hardly bear to be near himself when the evacuation took place. The treatment was followed by saline draught and by subnitrate of bismuth, with the result that in three days all pain ceased.

The dental practitioner meets with difficulties in that the patient does not consult him in regard to the general state of health, and he is compelled to put leading questions from external observations only. His opportunity is sometimes greater for a diagnosis than that of the medical man, as the patient is longer under his observation at close quarters, and he should insist that his patient go under treatment from the medical adviser before commencing any serious dental work.

The following may be considered among the causes of stomatitis: An overloaded stomach with indigestible food; too much iced-water at meal-time; exudations from chronic ulcers or abscesses mixing with food swallowed; excessive smoking; imperfect mastication; inflammation and ulceration of the tonsils or larynx; excess of uric acid; and last, though not least, pin-worms in both children and adults, often caused by an excessive meat diet.

The symptoms of the last-named cause of stomatitis are very marked,—twitching of the eyelids with a dulness of the pupils, muscular contortions of the face, sleepless nights, uncertain appetite, and grinding of the teeth. Recognizing these conditions in a child brought by her mother, accompanied by two sisters, I called attention to it, and the mother said her doctor pronounced it nervousness from study. Examination showed her teeth in perfect order, so I advised an examination of the excrement, when pinworms were found. I prescribed one grain of santonin every morning for a week, fasting, and on alternate nights five grains of extract of cascara. In three weeks the child lost all trace of trouble, and has continued in robust health.

Another case. A lady of about twenty-two years of age, whom I had attended for some years, came for relief of neuralgia. She said she was under treatment for stomach trouble. She was very weak, and nearly fainted in the chair. A searching look convinced me she was lacing very tightly. I told her of it, and prescribed a sedative of compound kino powder, ten grains twice a day, and some Rochelle salt. All pain and distress disappeared by the end of the week, and I do not think she will lace tightly again.

Nausea in impression-taking is very distressing, and the patient subject to this condition may often be benefited by timely treatment. A case of this kind baffled every dentist the lady visited, and she had to dispense with teeth on this account. She had been treated for stomatitis by many practitioners and at various hospitals without result. During gestation this condition was somewhat improved, but after the birth it increased to a violent extent, and ended in death, but the coffee-ground exudation just previous proved it to be a case of cancer of the pylorus.

Allow me to conclude by saying that I would never open a root for a patient suffering from constipation without first removing the ailment, as it may be termed, and administering in one-grain doses, three times a day for three or four days, sulphite of calcium to eliminate the stomatitis consequent on this condition, and which may lead to veritable poison.

